# Recycling of Plastic Waste in the Construction Industry

**DOI:** 10.3390/polym17091282

**Published:** 2025-05-07

**Authors:** Nancy Sakr, Mohamed AbouZeid

**Affiliations:** Construction Engineering Department, The American University in Cairo, New Cairo 11835, Egypt; mnagiba@aucegypt.edu

**Keywords:** energy resources, plastic wastes, recycled plastic, recycled concrete, construction industry, sustainability

## Abstract

This study underscores the importance of sustainable practices by exploring the utilization of recycled plastic within the global construction industry. Plastic recycling has emerged as a crucial strategy that aligns with environmental, social, and economic sustainability indicators. Currently, substantial volumes of plastic waste are either deposited in landfills or incinerated, neglecting the potential to harness its embodied energy and the energy consumed for producing virgin materials. A key advantage of plastic lies in its promising mechanical properties. Concrete mix design is fundamental to a wide range of construction applications, including brick walls, reinforced concrete slabs, and concrete pavements. Despite the adoption of recycled plastic in construction materials in various countries, its widespread implementation remains limited. This is primarily due to the scarcity of experimental research in this area and the absence of a robust waste management system. This research specifically investigates the reuse of two common types of plastic waste: polyethylene terephthalate (PET) and high-density polyethylene (HDPE) to mitigate plastic waste accumulation in landfills and enhance the performance of construction materials. The study investigates the use of recycled HDPE and PET as a replacement for coarse aggregates in concrete pavement mixtures. While recycled PET is more prevalent in concrete applications, recycled HDPE has demonstrated exceptional efficiency and durability. The recycling method used in this research is the mechanical recycling method due to its superior effectiveness in comparison with other methodologies. This research assesses the performance of recycled PET and HDPE in concrete pavement, aiming to diminish non-renewable energy consumption by 15–20%, curtail the carbon footprint by 15–30%, and decrease plastic waste in landfills by 20–30% compared to conventional concrete.

## 1. Introduction

The issue of Municipal Solid Waste (MSW) demands urgent global attention. As noted by World Data, the potential for repurposing materials beyond their original applications is significant. However, the sheer volume of waste produced, particularly plastic, poses a critical challenge. The United States, for instance, leads in per capita plastic waste production, generating an alarming 130.09 kg per person annually. This translates to over 42 million metric tons of plastic waste in 2016 alone, positioning the United States as the world’s largest plastic waste generator [1]. Current waste generation rates are exceeding landfill capacity, highlighting the pressing need for sustainable recycling solutions. Both developed and developing nations are actively seeking alternatives to traditional methods like landfill disposal and incineration due to escalating costs, logistical complexities, and adverse environmental consequences.

Recycling and upcycling are acknowledged as refined alternatives to conventional waste management strategies. According to the European plastic strategy, it is essential to reuse and recycle waste materials as much as possible to minimize waste disposal or incineration [2]. Recycling involves novel projects through techniques such as material extrusion printing and injection molding, specifically to generate items from plastic refuse [3]. These recycling processes can be categorized as mechanical, chemical, or thermal. Conversely, upcycling prioritizes the metamorphosis of used materials into superior-quality new products. A case in point is the design of sustainable seating solutions from discarded automobile tires or the fabrication of roofing tiles from reclaimed plastic [4].

Recent statistics from 2023 indicate that plastic packaging accounts for the majority (36%) of plastic production [5]. Of even greater concern is the breakdown of plastic waste management: 46% ends up in landfills, 22% becomes litter, 17% is incinerated, and only 15% is collected for recycling, with less than 9% ultimately recycled after accounting for losses [5]. MSW encompasses both organic waste, such as food waste, and inorganic waste, including wood, glass, metal, paper, and plastic. Plastic waste alone constitutes over 10% of global landfill content, lacking effective sorting mechanisms for recycling [6]. This complex issue underscores the global challenge of managing MSW effectively and sustainably. As noted, “Thermoplastic composites can be reshaped and are easier to recycle. They have a long shelf life and exhibit a high level of resistance to harmful chemical environments. As a result, they can be used as geopolymers in concrete composites, serving as an alternative to natural aggregates” [2].

While a heterogeneous array of plastic waste exists, a substantial proportion remains outside the ambit of significant recycling efforts. Each polymer exhibits discrete mechanical attributes that must be congruent with the intended utility of the reclaimed material. Moreover, the energetic cost inherent in the recycling process constitutes a salient consideration. To illustrate, polyethylene exists in two primary forms: low-density polyethylene (LDPE) and high-density polyethylene (HDPE). Each variant manifests unique mechanical characteristics that render it appropriate for specific applications. With a fusion point spanning from 120 to 160 °C, HDPE is particularly efficacious for concrete pavement applications in contrast to LDPE, which possesses a melting point within the range of 105 to 115 °C [4,7].

This research investigates the potential of utilizing a novel blend of recycled polyethylene terephthalate (PET) and recycled high-density polyethylene (HDPE) in concrete pavement applications. The specific blending ratios explored, in conjunction with established industry standards, offer a transferable methodology applicable to diverse plastic waste streams. This approach enables the assessment of requisite mechanical properties for various concrete applications within the construction sector. Furthermore, this research introduces a pioneering, adaptable framework capable of addressing a wide spectrum of plastic waste, thereby unlocking numerous avenues for effective waste migration. The originality of our material assessment lies in its integration of waste utilization within the construction industry, coupled with a systematic evaluation protocol based on the intrinsic attributes of each waste typology.

Plastics exhibit a wide range of properties, making them suitable for diverse applications. This article explores the experimental implementation of recycled polyethylene terephthalate (PET) and recycled high-density polyethylene (HDPE), emphasizing the latter, which is often sourced from flexible plastics like discarded containers. Recycled PET, commonly derived from used beverage bottles, is also examined [1]. PET is favored in recycling streams due to its desirable characteristics and ubiquitous presence, contributing to its frequent repurposing [1,2,3,4,5,6,7,8].

Polyethylene terephthalate (PET), a thermoplastic polymer in the polyester family, is renowned for its chemical, mechanical, and thermal resistance, as well as its dimensional stability [9]. Despite its versatility and advantages like durability and strength [1], PET is not biodegradable. Its widespread use stems from its favorable properties. High-density polyethylene (HDPE), another thermoplastic derived from petroleum, is considered a highly valuable plastic material. Characterized by its long chains of repeating monomer units (C_2_H_4_), HDPE exhibits a relatively low degree of branching compared to other polyethylene categories [10]. This structural feature contributes to its high tensile strength and elevated melting point. HDPE enjoys extensive application across diverse products, including plastic bottles, piping, and cutting boards, and commands over 34% of the global market. Known as “Type 2 plastic”, HDPE is employed in the production of thicker, more robust containers compared to those made from PET. Typical examples include milk jugs, motor oil containers, detergent bottles, and bleach bottles [8].

**Figure 1:**
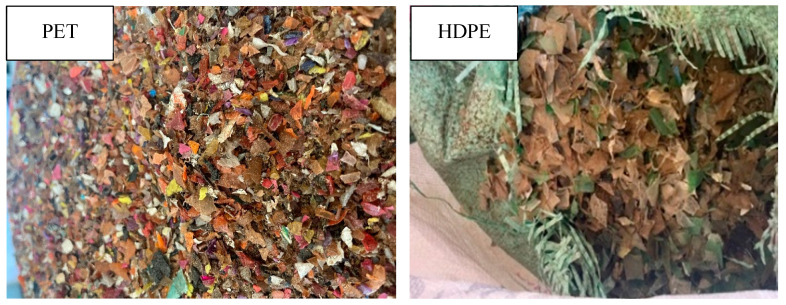
Unprocessed plastic waste before preparation.

The benefits of incorporating recycled PET and HDPE in construction commence with the financial advantages of decreasing waste deposited in landfills. Employing recycled plastic can result in substantial cost savings in the production of new construction materials [9]. The utilization of plastic waste curtails the quantity of construction materials needed for projects, as the plastic recycling procedure is approximately 10% less expensive than producing new materials without compromising the material’s properties. This has been thoroughly addressed in the ASTM Standard List (2023) [8]. Furthermore, this utilization strategy enhances the material’s characteristics when recycled plastic is incorporated into construction. Research and testing have demonstrated that recycled PET and HDPE either maintain or improve the material’s properties [10]. This occurs alongside a reduction in the construction industry’s energy requirements. A noteworthy global advantage is that development programs for plastic recycling in various countries allocate supplementary funding to promote the implementation of development plans [10]. Figure 1 illustrates shredded PET and HDPE plastic [11].

One compelling reason to prioritize plastic waste management is the correlation between high population density and substantial waste generation [12]. Densely populated areas are likely to produce greater solid waste, particularly in the absence of effective waste management infrastructure. To advance sustainability goals, integrating a circular economy model through the utilization of recycled plastics is crucial. This approach has the potential to yield a 50% reduction in greenhouse gas emissions originating from the plastic sector [10,11,12,13]. As previously indicated from the standpoint of plastics, a circular economy can be realized through a multifaceted strategy encompassing product redesign, enhanced post-consumer collection systems, the increased adoption of reusable plastic products, and innovative business models, alongside expanded recycling capabilities [13]. The traditional linear economic model, often referred to as “Cradle to Grave” [14], is unsustainable, as it culminates in waste accumulation in landfills due to inadequate processing. In contrast, the circular economy fosters a continuous cycle of resource utilization, epitomized by the “Cradle to Cradle” framework [14]. This approach aims to establish a perpetual product lifecycle, incorporating recycled materials to maintain a closed-loop system [10]. The European Commission has emphasized in its European Plastics in a Circular Economy Strategy that by 2025, 50% of composite waste and all plastic waste must be reused or recycled, with the target increasing to 55% by 2030. This initiative underscores the urgent need to explore advanced recycling alternatives that can help reduce energy consumption and improve material efficiency [15].

Synergistic collaboration between industries has the potential to yield significant progress in sustainability and energy conservation. Several nations have already adopted the use of recycled plastics in the production of pavement tiles, which can be processed into various sizes depending on the intended application [16]. Furthermore, plastic can effectively substitute coarse aggregates in the concrete mixture [17]. As noted, “The increasing preference for environmentally sound, cost-effective, and lightweight construction materials within the building sector has spurred a demand for research into methods that achieve these objectives while simultaneously benefiting the environment and adhering to material standards” [18].

A significant drawback of plastic recycling lies in its temporal displacement of the landfill problem, burdening future generations with the accumulated waste. The environmental persistence of microplastics, resulting from weathering and abrasion, further intensifies this issue. However, it is important to acknowledge that plastic has been an environmental challenge since the material’s inception. By adhering to sustainability principles, we can proactively investigate and implement more effective environmental solutions [19].

The significant contribution of plastics to climate change is another pressing concern. The vast majority of plastics are derived from fossil fuels, with only a negligible fraction originating from recycled sources.

Climate change is a multifaceted challenge impacted by numerous variables, including the methods employed in the production of construction materials and the disposal of solid waste [17,18,19].

Furthermore, sustainable design serves as a crucial strategy for attaining energy efficiency and fostering a healthier environment. This necessitates a delicate equilibrium between resource input and output. For example, mitigating the environmental impact and diminishing the carbon footprint of premium construction projects represent substantive progress toward achieving sustainability and the United Nations’ Sustainable Development Goals (SDGs) 2023 Agenda. The SDGs framework has been embraced globally, with the 17 goals outlining interconnected social, economic, and environmental sustainability indicators. Both developed and developing nations are called upon to engage in concerted action through this global partnership [20].

Concrete manufacturing is a notably energy-intensive process. A typical concrete mix comprises cement, aggregates (both coarse and fine), and water. This process generates significant emissions, particularly carbon dioxide. As noted, “Over a 50-year life span, embodied energy constitutes 45% of the total energy demand. The recycling potential ranges between 35% and 40% of this embodied energy” [21]. The embodied carbon in construction materials refers to the cumulative carbon dioxide emissions released during their production. This includes emissions throughout the entire construction lifecycle: from raw material extraction and transportation to the energy consumed by fixtures during a building’s operation and, finally, through demolition or ongoing maintenance. Table 1 details the embodied carbon values for various building materials. Of those mentioned, steel demonstrates the most carbon-intensive, followed by glass, timber, brick, and concrete. For perspective, brick production averages 0.7 kg CO_2_/kg, while concrete production averages 0.3 kg CO_2_/kg [22]. The primary carbon emission phases are the raw material sourcing and the concrete manufacturing processes [23]. Raw material origins vary: Cement, the binding agent, is derived from limestone and clay; coarse aggregates are obtained from crushed natural stone; and fine aggregates from gravel.

A key factor in incentivizing the use of recycled materials, and thus, reducing embodied carbon, lies in the lower embodied carbon footprint associated with these materials [23]; take, for example, the significant difference between virgin and recycled PET and HDPE plastics. Virgin PET carries an embodied carbon burden of 2.15 kg CO_2_ equivalent per kilogram, while recycled PET demonstrates a substantially lower value of 0.45 kg CO_2_ equivalent per kilogram. This translates to a 79% reduction in CO_2_ emissions, as evidenced by a 2017 study conducted by a major PET producer in Austria, when utilizing recycled PET over virgin material [22].

Beyond plastic, a wide array of waste materials can serve as viable substitutes for conventional coarse aggregates in concrete mixes [23] by leveraging the embodied energy EE values provided by databases such as the Inventory of Carbon and Energy ICE [22]. The overall embodied energy and, by extension, the embodied carbon of these modified concrete mixes can be accurately assessed based on the specific type of waste employed.

According to data from the Institute of Civil Engineers (ICE), each material possesses a unique embodied energy value, which is presented in Table 1. It is evident that cement has the highest embodied energy per kilogram, exceeding that of both aggregate and water. In addition, the production of cement generates the most substantial carbon dioxide emissions when compared with aggregate production.

The nexus between elevated energy consumption, embodied carbon dioxide, carbon footprint metrics, reliance on fossil fuels, and plastic manufacturing is undeniable. The common thread linking these elements is the energy invested in production and the pursuit of sustainability [22]. Each process demands a discrete energy expenditure to generate a refined, processed, or environmentally sustainable material. True sustainability is realized through adherence to established sustainability benchmarks, ultimately safeguarding our natural and nonrenewable energy reserves [23].

This research employs laboratory experiments to rigorously evaluate and compare the mechanical properties of conventional and recycled plastic concrete, focusing on compressive and tensile strength, as well as fire resistance. A comparative analysis of these properties is conducted. Furthermore, a sensitivity analysis, derived from the experimental findings, serves as a model for the wider application of recycled materials in construction. The results of the sensitivity analysis for recycled pavement concrete demonstrate that the recycled plastic concrete exhibits comparable mechanical properties to conventional mixes. In certain instances, the compressive strength of the recycled plastic concrete even surpasses that of its conventional counterpart [17,18,19,20,21,22,23,24,25]. While the incorporation of recycled plastic in concrete is prevalent in industrialized nations, awareness and adoption remain limited in other regions.

## 2. Materials and Methods

Recycled polyethylene terephthalate (PET) is a widely utilized and economically advantageous plastic. In contrast, recycled high-density polyethylene (HDPE), a thermoplastic polymer derived from petroleum, is distinguished by its exceptional durability and chemical resistance, rendering it suitable for outdoor applications, as previously documented [26]. Furthermore, HDPE is a recyclable and sustainable material, facilitating its reuse in diverse applications. Notably, the comparatively lower utilization of recycled HDPE relative to PET contributes to its enhanced cost-effectiveness and particular viability.

Given the primary objective of this investigation, which is to integrate plastic into the construction sector, it is imperative to consider the various factors that influence concrete performance and quality. These factors can be categorized as dependent and independent variables, which are the controlled parameters within the experimental framework (e.g., cement, coarse aggregates, water, plastic additives, and sand), which directly affect the dependent variables, such as concrete strength and durability.

### 2.1. Experimental Procedure

**Figure 2:**
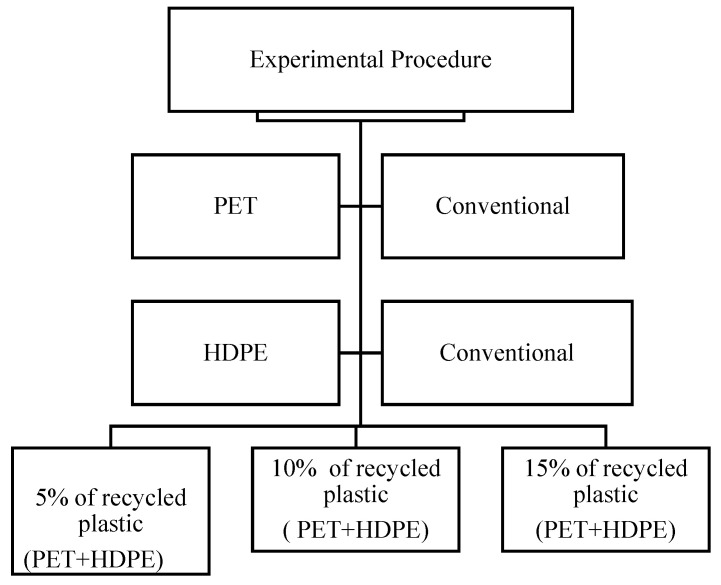
Experimental procedure.

The experimental procedure is divided into two main parts. The first part is the conventional testing for the concrete mix, and the second part is comparing the conventional concrete mix with the recycled concrete (PET and HDPE). Figure 2 shows the breakdown of the experimental procedure.

### 2.2. Preparation of Waste Material

**Figure 3:**
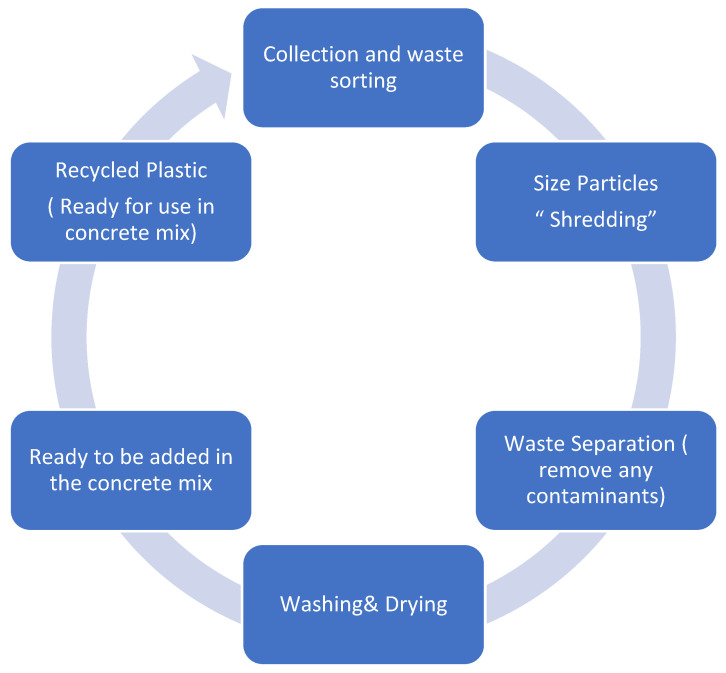
Material waste preparation.

**Figure 4:**
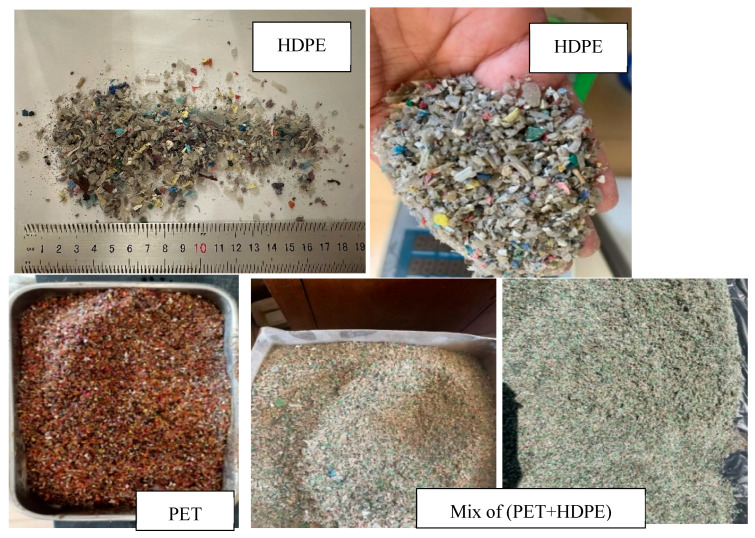
Stages of recycled plastic preparation.

**Figure 5:**
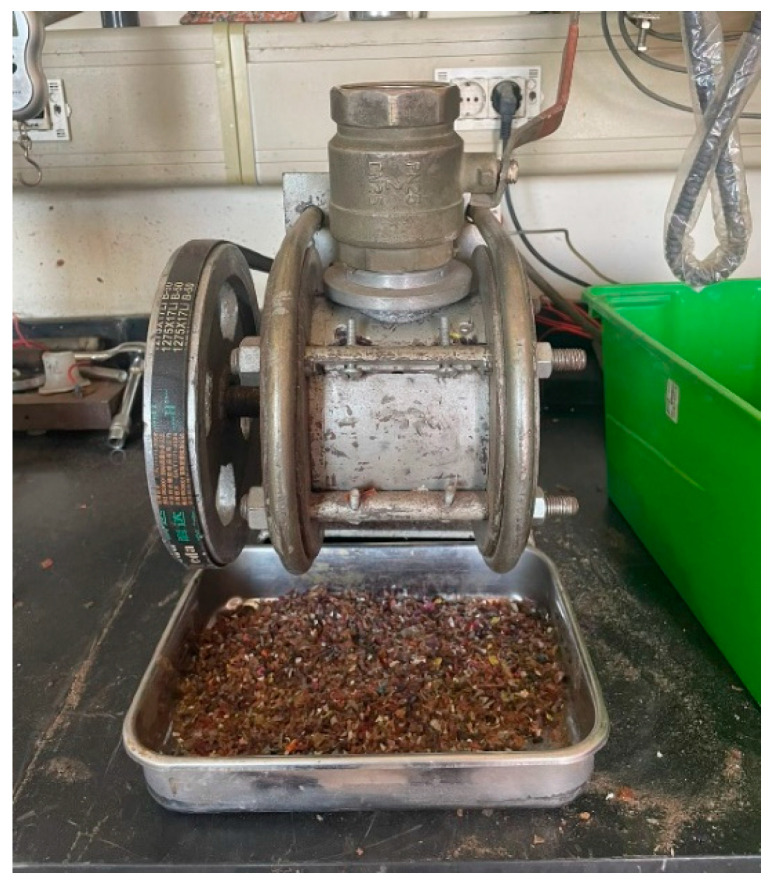
Plastic Shredding Machine for recycled PET.

The process of preparing plastic waste can undergo variations depending on the specific quality and source of the waste. It is crucial to acknowledge that the quality of plastic waste can exhibit fluctuations with each acquisition from garbage collectors and suppliers. For instance, during our research, we encountered two batches of plastic waste obtained from a supplier, and we observed that the first batch contained fewer contaminants than the second. This disparity in quality adversely affected the properties of the concrete we were utilizing, as the other materials and waste were contaminated with plastic. Consequently, to prevent the creation of non-binding concrete and any potential experimental failures, it is imperative to prepare the plastic before utilizing it as a material, as shown in Figure 3. The sequential steps for preparing plastic waste are as follows:

1. The process of incorporating recycled plastic into concrete begins with sourcing high-quality plastic waste from reputable suppliers. This ensures the material is free from undefined contaminants and that the waste is untreated, crucial for maintaining the integrity of the concrete.
2. The plastic undergoes multiple stages of shredding, as depicted in Figure 4 and Figure 5 by (Plastic shredding equipment machine, construction materials lab, AUC, Cairo, Egypt). This meticulous process facilitates optimal binding within the concrete matrix. Alternatively, sieving can be employed to achieve a specific particle size distribution. Once this is completed, the plastic is ready for storage and subsequent use in concrete mix designs.
3. A crucial step is sieving the shredded plastic to remove impurities. This prevents contaminants like dust and foreign particles from compromising the structural integrity of the final concrete product.
4. Following this, the plastic is thoroughly washed to eliminate any residual dust or debris that might interfere with the concrete mix. Figure 4 illustrates the various stages of this preparation process.
5. The washed plastic is then dried meticulously to ensure no residual moisture remains. This is essential for achieving optimal binding and reliable laboratory test results. Drying is typically accomplished by spreading the shredded plastic particles on a large surface.
6. Finally, the prepared recycled plastic is integrated into the concrete mix design within the concrete mixer.

### 2.3. Mechanical Recycling

There are three primary methodologies for recycling: mechanical, chemical, and thermal. This research employs mechanical recycling, selected for its lower energy consumption compared to the other methods. Within mechanical recycling, two distinct techniques exist: cold and hot processing. This study specifically utilizes the cold processing technique. It is noteworthy that: “In some regions, mechanical recycling accounts for 22% of recycled plastic, chemical (feedstock) recycling for 3%, and energy recovery (combustion) for 60%” [27].

**Figure 6:**
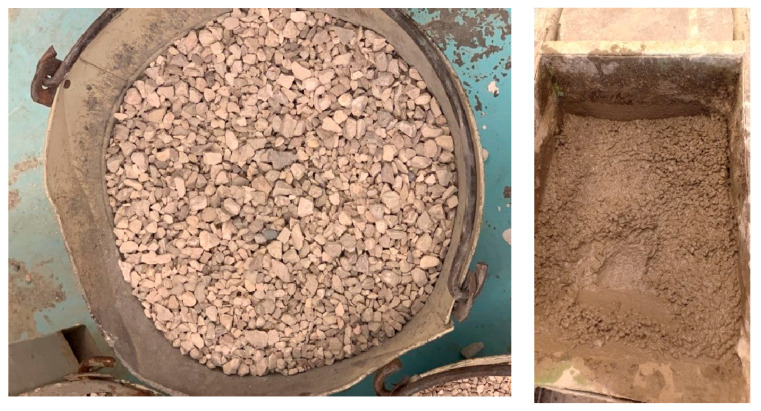
Coarse aggregates—Sieve #14 and shredded plastic—Sieve #30.

To achieve optimal proportions for concrete mix design, comminuted plastic necessitates sieve analysis using a grading machine. This step is critical as the particle size distribution of the plastic waste directly influences the mechanical properties of the concrete, including binding capacity and compressive strength. Similarly, coarse aggregate grading also undergoes sieve analysis, as the required aggregate size varies depending on the intended application of the concrete mix, such as paving or reinforced concrete. For instance, the concrete mix formulated for pavement in this study utilized a number 14 sieve grading for coarse aggregates with a sieve opening size of 14 mm and a number 30 sieve grading for shredded plastic (PET and HDPE) with a sieve opening size of 6 mm, as illustrated in Figure 6 [28]. The sieve openings conform to US standards, employed for both the coarse aggregate and the shredded plastic in the experiment, with each material assigned a specific grading number.

In addition, the experimental protocol was inherently shaped by a series of constraints that emerged during the laboratory investigation. Despite meticulous procedures and precautions during experimentation, several limitations were encountered.

A primary obstacle was the procurement of consistently high-grade recycled PET and HDPE, a challenge stemming from polymer degradation and contamination despite the widespread availability of plastic waste.

Stringent temperature control was also essential, with a maximum threshold of 30 °C, to mitigate the detrimental effects of heat on material expansion and strength.

The potential need for chemical admixtures to optimize concrete workability was considered. The selection of specific admixtures, such as water reducers, retarders, or accelerators, was contingent upon observed deficiencies during the mixing process, with standard dosages typically falling within the 0.2% range by mass of cement.

### 2.4. Future Recommendations for Waste Material Preparation

**Figure 7:**
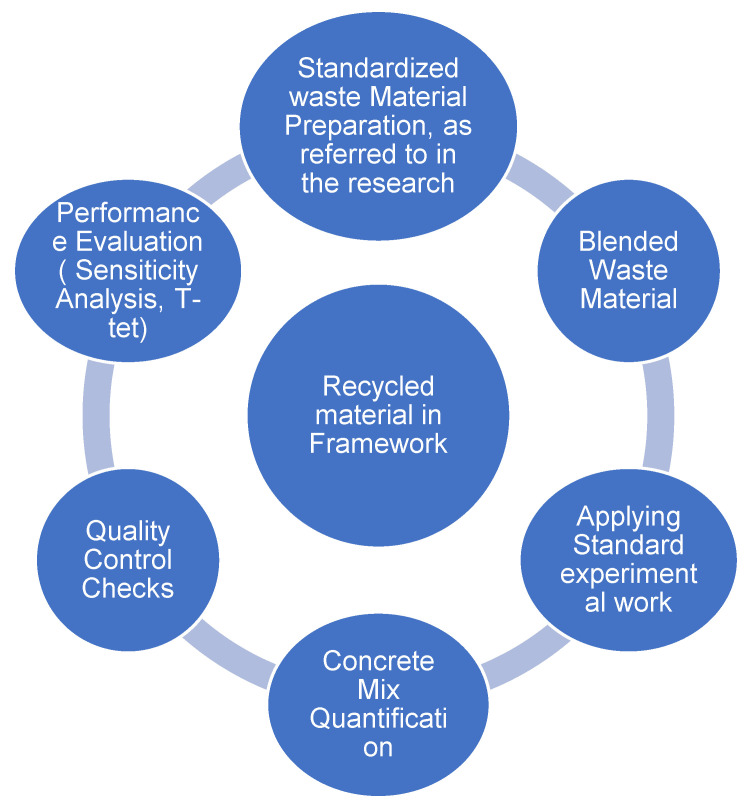
Recycled material framework.

- Ensuring the reliability of laboratory results necessitates rigorous and consistent quality control throughout all experimental phases, as shown in Figure 7. To achieve accurate and dependable outcomes, strict adherence to standardized quantities and procedures is paramount. Enhancing the quality control of concrete mix preparation specifically requires meticulous attention to the following key aspects:
- Optimal storage of constituent materials (cement, sand, and aggregates) to prevent degradation and maintain material integrity.
- Strict procedural adherence, precluding deviation from established protocols by laboratory technicians without explicit authorization from supervising engineers. Unilateral adjustments to mix proportions can compromise experimental validity.
- Precise gravimetric measurement of all constituent materials, ensuring adherence to established mix design calculations and relevant industry standards.
- Accurate aggregate gradation through meticulous sieving, recognizing that variations in sieve size distribution directly influence the binding properties and ultimate strength of the concrete mix.
- Comprehensive documentation of all laboratory outputs is essential to mitigate ambiguity and human error. Furthermore, the creation of a detailed spreadsheet, encompassing all inputs, outputs, test results, applied equations, and relevant standards’ references, is paramount for rigorous result verification against utilized quantities and the generation of informative graphical representations.

## 3. Results

### 3.1. Experimental Work

Formatting of Mathematical Components.

1. Specific gravity of water = 1
2. Specific gravity of cement = 3.15
3. Splitting tensile strength: σ = 2P/(π d L)
4. fc = Load/Area
5. Compressive strength: Cube compressive strength = 1.25 × Cylinder compressive strength.
6. fc = Load/Area
7. Mass (kN/m^3^) = ((Unit weight × Volume)/9.81)
8. Unit weight of fresh concrete kg/m^3^: (weight of concrete − the weight of an empty container)/(weight of water − weight of an empty container) × 1000.
9. Concrete permeability: The coefficient of permeability is measured in m/sec; it is a governing factor for determining durability and quality.
10. Unpaired *t*-test: $t=(\frac{\left(\bar{x},-{\bar{x}}_{2}\right)}{\surd \frac{S_{1}^{1}}{n_{1}}+\surd \frac{S_{2}^{2}}{n_{2}}})$ (x: Sample means; n: Sample sizes; s: Sample variance)
11. [Water cement (kg/m^3^)/(specific gravity of water) + Cement content ((kg/m^3^))/Specific gravity of cement + Coarse aggregate content ((kg/m^3^))/Specific gravity of coarse aggregates + Fine aggregate content (kg/m^3^))/Specific gravity of fine aggregates) + 1 − (2% air voids) = 1000]

**Figure 8:**
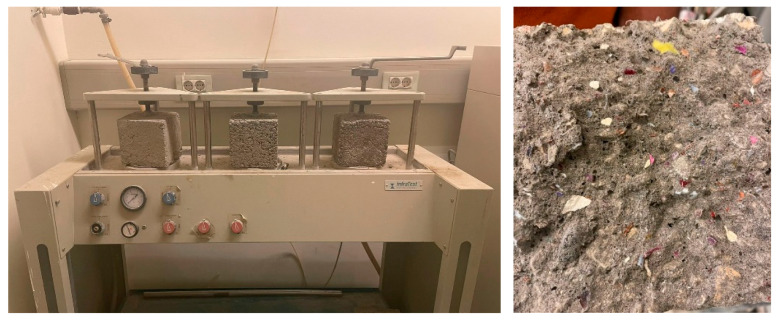
Concrete block cross-section and during the water permeability test.

**Figure 9:**
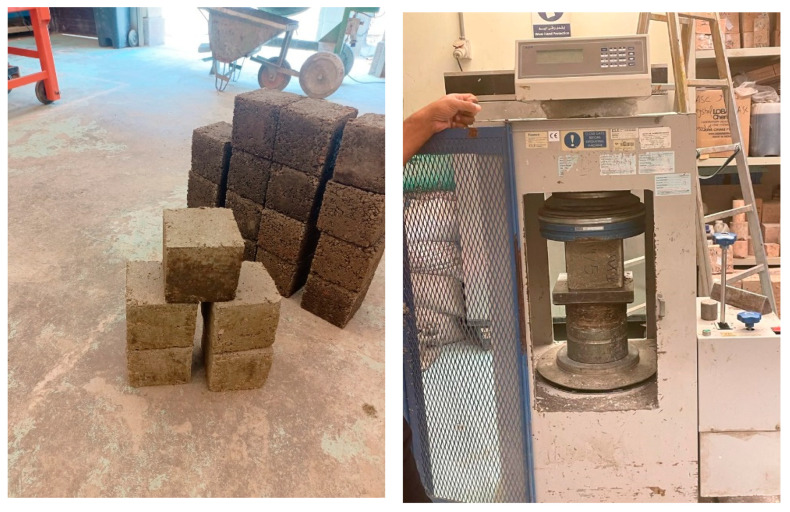
Concrete cubes and the compressive strength test.

The recycled plastic is divided into three tests: PET, HDPE, and a blend of PET and HDPE in the same concrete mix design. Each concrete mix was experimented with at different percentages (5%, 10%, and 15%). It is important to experiment with the recycled plastic concrete cubes’ chemical durability and water permeability, as shown in Figure 8, because sulfur attacks and water permeability are the limitations that should be measured in recycled plastic concrete pavement. The water permeability test is conducted using (InfraTest, Water permeability machine, Waldkraiburg, Germany) Additionally, exposure to fire or high temperatures is another limitation that should be measured on different levels; Figure 9 shows the concrete blocks during fire exposure.

To achieve the most appropriate concrete mix design for pavements, the American Concrete Institute (ACI) standards offer helpful tables that take into account functionality and location. Table 2 outlines the standards for the concrete mix design and pavement specifications, including a water–cement ratio of 0.48 and a target strength of 30 to 35. For one cube of 0.15 × 0.15 × 0.15 m, prior to conducting experimental work on a concrete mix design, it is important to consider certain guidelines and recommended prescriptions for fresh concrete testing [15,16,17,18,19,20,21,22,23,24,25,26].

Guidelines for concrete mix design preparation [15]

- A minimum of three concrete cubes for each experiment are prepared with a volume of 0.15 × 0.15 × 0.15 m^3^.
- The unit weight should be between 2100 and 2400 kg/m^3^, as per IS 456:2000 [15].
- It is mandatory that the temperature be less than 37 °C.
- The air content in an un-air-entrained mix should not exceed 3%, as this will decrease its compactness and result in a weak concrete mix.
- Fresh concrete testing should be well-measured, including slump, temperature, mass, and air content tests. The same applies to hardened concrete: compressive and tensile strength tests [29].
- Table 2 shows the ASTM standards for the testing results and the needed mechanical properties according to the functionality of the concrete mix.
- Adhering to ACI standards, the concrete mix design intended for pavement blocks must satisfy a defined range of performance outcomes. The material specifications for concrete pavement should be outlined as follows [10]:
  o W/C ratio = 0.48
  o M.S.A = 25 mm
  o Slump ranges between 1 and 3 cm
  o Target strength ranges between 30 and 35 MPa.

**Table 2:** ASTM standards for concrete mix design—mechanical properties [9,10,11,12,13,14,15,16,17,18,19,20,21,22,23,24,25,26,27,28,29,30].

Material Properties	ASTM Standards for Concert Pavement
Slump	ASTM C 143 [30]
Unit weight	ASTM C 138 [31]
Air content	ASTM C231-97 [32]
Compressive strength	ASTM C936/C936M, BS 1881 [33]
Fire resistance	ASTM E.119 [34]
Chemical durability	ASTM C1202 [35]
Water permeability	CRD-C 163-92 [36]
Chemical admixtures	ASTM C 494 [37]

**Figure 10:**
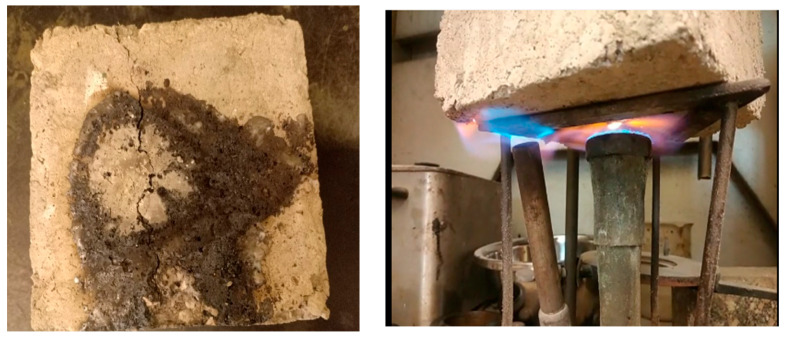
Concrete block fire resistance test—30 min.

Table 3 illustrated the standardized concrete quantities per cube when calculating the conventional concrete mix and the plastic waste percentage. Table 4 displays the average compressive strength and tensile strength for each percentage of recycled plastic used in the concrete mix. The first mix is the conventional concrete mix design for pavement blocks. However, the other three mixes contain recycled plastic in the concrete mix, ranging from 5% to 15%, as shown in Figure 10. The tests of strength are measure by the universal testing machine (ELE machine, construction materials lab, AUC, Pudong, Shanghai, China). The recycled plastic quantity is substituted for the coarse aggregate quantity in the mix to measure the best material properties by the plastic percentage in the concrete mix [18,19,20,21,22,23,24,25,26,27,28,29,30,38]. As illustrated from the concrete mix design quantities used in the experiments, the mix quantities are nearly the same, except for the coarse aggregates and the plastic percentage substituted quantities.

### 3.2. Experimental Findings

**Figure 11:**
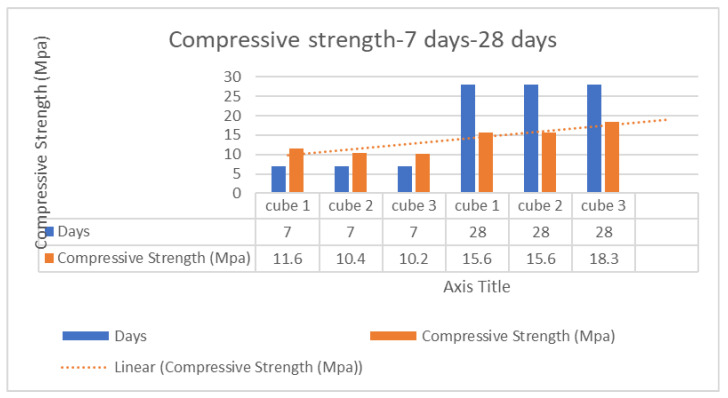
Compressive strength progression using recycled (PET + HDPE) concrete.

**Figure 12:**
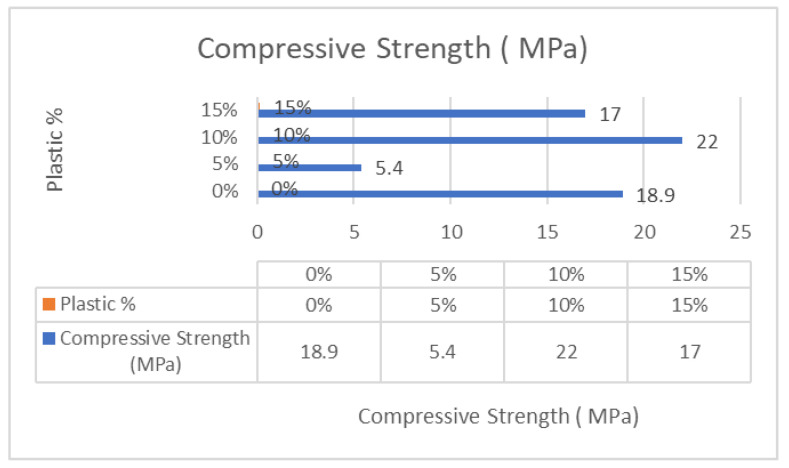
Compressive strength—28 days.

**Figure 13:**
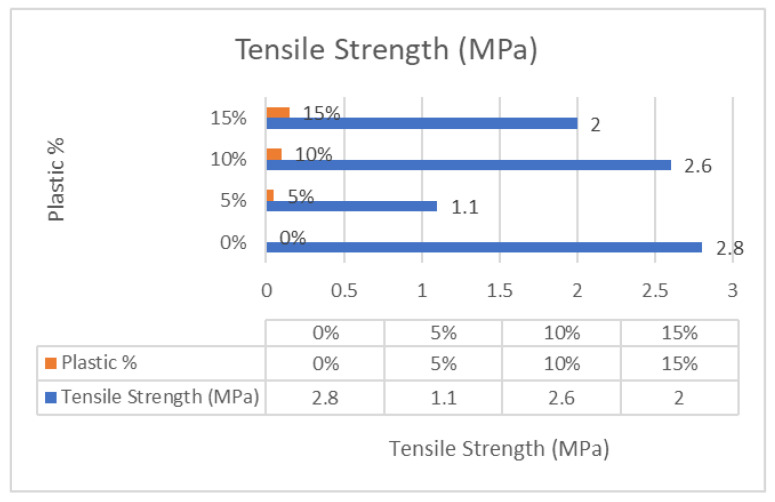
Tensile strength—28 days.

Based on the results of previous experiments, charts were created to analyze and compare the various concrete mixes. Figure 11 displays a chart showing the compressive strength from 7 days to 28 days. Also, it was discovered that incorporating 10% recycled plastic into the concrete mix produced the best compressive strength, with Mix-PL10 reaching 22.0 MPa compared to 19.0 MPa for conventional concrete, as shown in Figure 12. These findings demonstrate that recycled plastic concrete can achieve similar or even greater strength than conventional concrete. Conversely, the lowest compressive strength was observed in the concrete mix containing only 5% recycled plastic, which is also reflected in the tensile strength chart presented in Figure 13. The compromised mechanical integrity observed in the 5% recycled concrete composite can be ascribed to the suboptimal dimensions of the shredded plastic and its limited cohesive properties. Furthermore, the purported cost-effectiveness of this particular formulation does not exert a significant impact on the comprehensive economic evaluation when juxtaposed with mixtures incorporating a greater proportion of plastic content.

**Figure 14:**
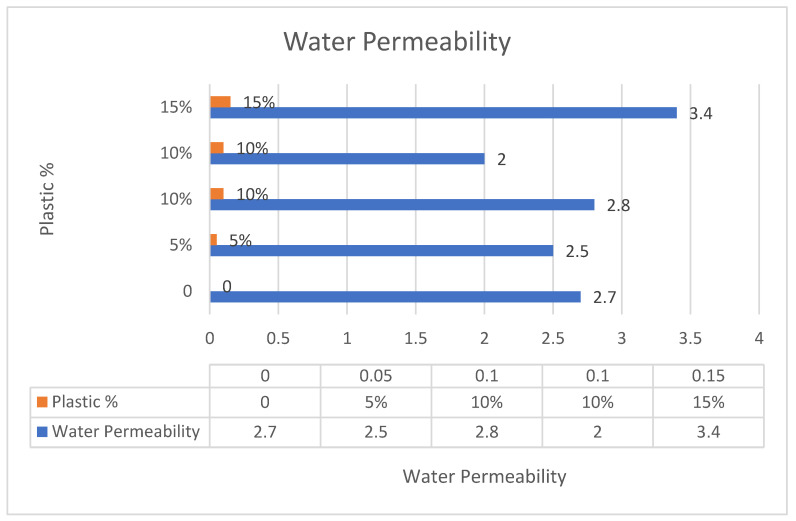
Water permeability in the concrete mix.

Upon the water permeability test, it was found that the best result of water permeability was when the plastic ranged between 10% and 15% of the coarse aggregates in the concrete mix, as shown in Figure 14. A drop occurred in one of the experiments containing 10% recycled plastic because of the poor quality of plastic waste and the usage of unwashed shredded plastic. However, the test was repeated and succeeded after maintaining the quality control guidelines, as shown in the chart.

## 4. Discussion

The workability of the concrete mix is a crucial determinant of the final concrete properties. Concrete cubes, as illustrated in Figure 5, should be well compacted to eliminate any air voids within the mix. Intriguingly, the properties of the concrete mix incorporating recycled plastic were found to be similar to those of conventional concrete.

According to ACI standards, the designated properties of the constituent materials are expected to align with established values. These values are crucial for ensuring the intended performance of the concrete mixture and the performance attributes identified through laboratory testing. To illustrate, the Slump test, a measure of the concrete workability, should yield results within a range of 1 to 3 cm to mitigate the risk of producing structurally deficient concrete. Furthermore, the specific target strength for concrete pavement should be predicated on a standard average ranging from 30 to 35 MPa [26].

Each concrete block utilized seven small mineral water plastic bottles. In certain experiments, admixtures were incorporated to enhance the workability of the mix. A consistent finding across all experiments was the necessity of a higher water percentage than in conventional mixes to achieve optimal workability. This increase in the water ratio amounted to approximately 5% of the standard water content.

Regarding the hardened concrete experiments, water permeability and fire resistance are crucial parameters to assess, as they directly relate to the limitations of plastic performance within the concrete mix. The most favorable water permeability results were observed when plastic content reached 15%, and 10% performed second best, as depicted in Figure 6. Similarly, comparable fire resistance was noted between mixes containing between 10% and 15% of the plastic in the mix. However, the mix with the 5% recycled plastic attained the weakest result, as shown in Figure 7. The fire testing shown in Figure 7 revealed that after half an hour of exposure to fire, the weight of the 15% recycled plastic in the mix before exposure was 7.34 kg and after was 7.30 kg. Notably, the concrete block did not reach the melting point within this timeframe. Nevertheless, further experimentations are imperative to ascertain the precise melting point and develop any mitigation strategies.

### Experimental Challenges

During the experimental work, several challenges emerged while preparing the concrete mix that incorporated recycled plastics. It became evident that the mix required more water than the initially calculated amount or what is typically used in conventional mixes. The results from the slump test, along with the observed consistency of the concrete, indicated that additional water was necessary to achieve optimal workability. Moreover, it was essential to increase the quantity of sand to ensure better binding of the materials. Consequently, specific protocols should be adhered to when conducting these experiments.

Unpaired *t*-test and Cost Analysis

**Figure 15:**
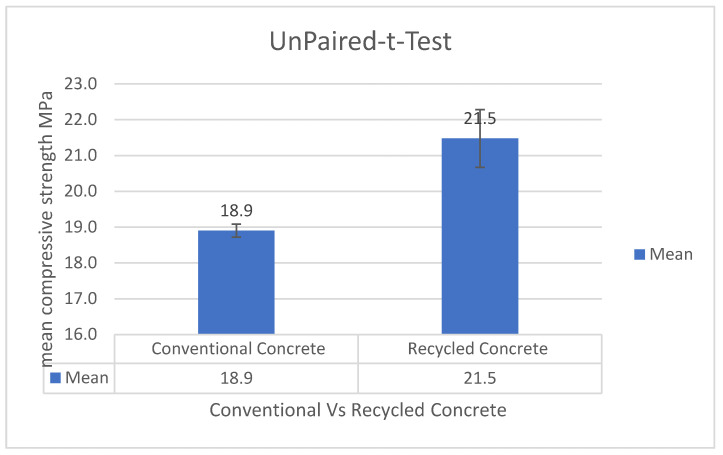
Conventional concrete vs. recycled plastic concrete *t*-test.

- *t*-test-unpaired is conducted by Excel to calculate the *p* value. The compared values are the compressive strength results between conventional concrete and recycled plastic concrete (average between compressive strength values for PET and HDPE).
- The *t*-test in Figure 15 shows that recycled plastic concrete performs differently from conventional concrete. The mean value on the *Y*-axis represents the difference between conventional and recycled concrete, as indicated on the *X*-axis. The error bars illustrate the significance of this comparison.
- Figure 15 shows the graphical presentation of the *t*-test implementation, showing the significant satisfaction when using recycled plastic in the concrete mix. The values are shown in Table 5.
- The exact *p* value equals 0.02, which means that there is a significant difference between the two results of compressive strength.

A cost analysis is implemented on a case study of a plot area of 250 m^2^. The number of concrete cubes is calculated accordingly, and then the cost per kilogram and item, as shown in Table 6. A cost comparison is applied between the conventional and recycled plastic to get the percentage of savings, as shown in Table 7. By this exercise, the cost efficiency is proven to meet the sustainability pillars.

**Table 5:** *t*-test values.

*t*-Test: Two-Sample Assuming Unequal Variances		
Compressive strength	Conventional Concrete	Recycled Concrete
Mean	18.9	21.5
Variance	0.2	3.9
Observations	6.0	6.0
Standard error	0.2	0.8
Hypothesized Mean Difference	0.0	
df	5.0	
t Stat	−3.1	
P(T ≤ t) one-tail	0.0	
t Critical one-tail	2.0	
P(T ≤ t) two-tail	0.0	
t Critical two-tail	2.6	

## 5. Conclusions

In summary, the incorporation of recycled plastic waste into concrete mix designs for pavement applications presents a highly efficacious strategy, yielding demonstrably superior mechanical properties. The compressive and tensile strength outcomes frequently equal or surpass those achieved with conventional concrete formulations. Both recycled polyethylene terephthalate (PET) and high-density polyethylene terephthalate (HDPE) demonstrate remarkable performance and durability characteristics. Further research endeavors should prioritize recycled HDPE due to its advantageous mechanical properties of compressive and tensile strength, greater accessibility compared to PET, and a cost reduction of 30–50% relative to recycled PET.

This methodology not only significantly curtails landfill waste and incineration but also contributes to a reduction in energy consumption within the construction sector, thereby establishing itself as a definitive sustainable solution that addresses environmental, economic, and societal imperatives. Based on the empirical findings, the principal outcomes are as follows:

- The optimal incorporation ratio for recycled plastic within concrete matrices is determined to fall between 10% and 15%, as this range facilitates the attainment of the most desirable material properties.
- The development of a standardized protocol for a recycled material framework, which incorporates solid waste into concrete mix design, can substantially enhance the rigor and efficacy of subsequent experimental investigations.
- The incorporation of both recycled PET and HDPE into concrete mixtures yields comparable performance and material characteristics. It is encouraging to note a growing global focus on sustainability across various communities and nations, as evidenced by India’s successful implementation of similar initiatives.

Based on the innovative integration of recycled PET and HDPE as a unified recycled plastic component within concrete pavement mix design, an innovative framework is proposed to advance the construction and solid waste management industries toward more effective waste mitigation strategies.

### Recommendations

Ongoing research in this domain is crucial, as it provides essential insights and academic references for future endeavors. Collaborations and partnerships focused on sustainable concrete research are becoming more common, presenting new opportunities for exploration. Traditional methodologies can be refined to foster the development of more sustainable communities. The construction industry in Egypt, in particular, could hold significant potential, contingent upon improvements to the waste management system. Addressing these challenges and raising public awareness are essential for progress.

- Lack of governmental regulations to let people apply the approach, even with incentives.
- Lack of awareness among people.
- Lack of industry guidelines, including recycling as a sustainable approach instead of the traditional approaches.

## Figures and Tables

**Table 1:** Embodied energy and carbon dioxide emission values [22].

Ingredient	Embodied Energy (MJ/kg)	Carbon Dioxide (CO_2_/kg)
Cement	4.6	0.83
Aggregate	0.1	0.005
Water	0.20	0

**Table 3:** Standardized concrete quantities per cube in compliance with the plastic waste %.

Concrete Mix per One Cube (0.15 × 0.15 × 0.15 m)	Water/kg/m^3^	Cement/kg/m^3^	Sand/kg/m^3^	Aggregates/kg/m^3^	Plastic % from Coarse Aggregate
Mix 1	180.0	375.0	533.2	1242.0	0
Mix-PL0.05	180.0	375.0	533.2	1180.0	5%
Mix-PL0.10	180.0	375.0	533.2	1118.0	10%
Mix-PL0.15	180.0	375.0	533.2	1055.7	15%

**Table 4:** Concrete mix design properties.

Tests (Conventional)	Slump (cm)	Temperature (°C)	Unit Weight (kg/m^3^)	Mass (kN/m^3^_)_	Air Content	Compressive Strength (MPa)
Mix 1-Cube 1	2.0	24.4	2501.6	24.5	5.0	18.3
Mix 1-Cube 2	2.0	24.4	2501.6	24.5	5.0	18.7
Mix 1-Cube 3	2.0	24.4	2501.6	24.5	5.0	19.6
**Tests (10% Plastic)**	**Slump (cm)**	**Temperature (** **°C)**	**Unit Weight (kg/m^3^)**	**Mass (kN/m^3^)**	**Air Content**	**Compressive Strength (MPa)**
Mix-PL0.10-Cube 1	6.0	28.7	2363.0	23.2	5.5	20.0
Mix-PL0.10-Cube 2	6.0	28.7	2363.0	23.2	5.5	21.8
Mix-PL0.10-Cube 3	6.0	28.7	2363.0	23.2	5.5	24.0
**Tests (15% Plastic)**	**Slump (cm)**	**Temperature (** **°C)**	**Unit Weight(kg/m^3^)**	**Mass (kN/m^3^)**	**Air Content**	**Compressive Strength (MPa)**
Mix-PL0.15-Cube 1	1.0	26.0	3133.0	30.7	7.2	15.6
Mix-PL0.15-Cube 2	1.0	26.0	3133.0	30.7	7.2	16.9
Mix-PL0.15-Cube 3	1.0	26.0	3133.0	30.7	7.2	18.2

**Table 6:** Cost Analysis for the Concrete Mix Design.

Project 1			
Area (m^2^)	250	Cost (kg)	Cost per item (USD)
Number of cubes	11,112		
weight of water/kg	7763	0.04	305.32
weight of cement/kg	16,173	0.06	954.14
PET/kg	5356.55	0.24	1264.04
C.A/kg	48,203	0.69	33,181.15
F.A/kg	23,000	0.57	13,567.47
Total project cost			49,491.01
**Project 1**			
Area (m^2^)	250	Cost (kg)	Cost per item (USD)
Number of cubes	11,112		
weight of water/kg	7763	0.04	305.32
weight of cement/kg	16,173	0.06	954.14
HDPE/kg	6000	0.12	63.48
C.A/kg	48,203	0.69	33,328.55
F.A/kg	23,000	0.57	13,627.74
Total project cost			48,856.18

**Table 7:** Cost Comparison Between the Conventional and Recycled Plastic Concrete.

Project Area (m^2^)				
PET Percentage	500.00 m^2^	750.00 m^2^	1000.00 m^2^	% of Saving
	Cost per USD			
-	103,800	155,700	207,600	-
5%	101,000	152,000	202,800	2.3%
10%	99,000	148,000	198,000	5.0%
15%	985,400	145,000	193,000	7.0%
**Project Area (m^2^)**				
HDPE Percentage	500.00 m^2^	750.00 m^2^	1000.00 m^2^	% of Saving
	Cost per USD			
-	103,800	155,700	207,600	-
5%	100,700	151,100	201,500	3.0%
10%	97,700	146,500	195,400	6.0%
15%	94,600	142,000	189,200	9.0%

## Data Availability

Data are contained within the article.

## Abbreviations

The following abbreviations are used in this manuscript:

PL0. (num)	plastic percentage in the concrete mix
PET	polyethylene terephthalate
HDPE	high-density polyethylene
LDPE	low-density polyethylene
ASTM	American Society for Testing and Materials
M.S.A	maximum size of aggregate
ACI	American Concrete Institute
